# Selenium Analysis and Speciation in Dietary Supplements Based on Next-Generation Selenium Ingredients

**DOI:** 10.3390/nu10101466

**Published:** 2018-10-09

**Authors:** Diana Constantinescu-Aruxandei, Rodica Mihaela Frîncu, Luiza Capră, Florin Oancea

**Affiliations:** 1 National Research & Development Institute for Chemistry and Petrochemistry ICECHIM, 202 Splaiul Independentei, 060021 Bucharest, Romania; luizacapra@yahoo.com; 2 INCDCP-ICECHIM Calarasi Subsidiary, 7A Nicolae Titulescu St., 915300 Lehliu Gara, Romania; icechim.calarasi@gmail.com

**Keywords:** selenium, analysis, speciation, next-generation supplements, selenium nanoparticles, zerovalent selenium, selenium polysaccharides

## Abstract

Selenium is essential for humans and the deficit of Se requires supplementation. In addition to traditional forms such as Se salts, amino acids, or selenium-enriched yeast supplements, next-generation selenium supplements, with lower risk for excess supplementation, are emerging. These are based on selenium forms with lower toxicity, higher bioavailability, and controlled release, such as zerovalent selenium nanoparticles (SeNPs) and selenized polysaccharides (SPs). This article aims to focus on the existing analytical systems for the next-generation Se dietary supplement, providing, at the same time, an overview of the analytical methods available for the traditional forms. The next-generation dietary supplements are evaluated in comparison with the conventional/traditional ones, as well as the analysis and speciation methods that are suitable to reveal which Se forms and species are present in a dietary supplement. Knowledge gaps and further research potential in this field are highlighted. The review indicates that the methods of analysis of next-generation selenium supplements should include a step related to chemical species separation. Such a step would allow a proper characterization of the selenium forms/species, including molecular mass/dimension, and substantiates the marketing claims related to the main advantages of these new selenium ingredients.

## 1. Introduction

Selenium was shown to be an essential trace element for humans. It got its name after Selene, the Greek goddess of the moon, and, like the moon, it has two opposite sides [1]. The bright side is related to its beneficial effects, for prevention and/or (adjuvant) treatment of various human and animal diseases (e.g., cardiovascular diseases, several types of cancers, and immune disorders) [2]. However, for the same disease, depending mainly on its exposure dose, there is also a dark side of selenium, related to disease promotion (e.g., type 2 diabetes) [3]. Selenium intake at the recommended level assures the balanced expression of bioactive selenoproteins, which act mainly as oxidoreductases, redox signal regulators, or for thyroid hormone activation [4]. However, even in the case of a moderate supranutritional intake, overexpression of selenium enzymes and/or excess formation of bioactive selenium metabolites determine pathophysiological effects [5].

A typical example is the complex relationship between selenium and cancer. Too low selenium intake caused high incidence of rectum, large intestine, ovary, breast, prostate, and lung cancers [6]. The lack of protection against DNA mutations, protection which is usually exerted by seleno-oxidoreductases, was involved in this high cancer incidence [7]. Selenium applied as selenium yeast at a supranutritional level of 200 μg/day determined a significant reduction in prostate, colon, and lung cancers [5]. The supranutritional level of selenium determines formation of selenium metabolites, such as seleniumdiglutathione, that activate apoptosis of transformed cells [5]. However, once the cancer cells are established, the supranutritional level and its associated increased expression of the antioxidant selenoenzymes protect the active cancer cells against apoptosis induced by the higher level of reactive oxygen species [7]. Moreover, when the daily intake is higher than the recommended dose, the pro-oxidant effects of inorganic Se have mutagenic effects and determine liver tumors [8]. Expression of several selenoproteins with anti-inflammatory and antioxidant effects induced by inorganic Se ingested at optimal doses may help prevent liver carcinogenesis [9,10].

A complex relationship and a U-shaped relationship between dose and disease were also demonstrated for type 2 diabetes. Epidemiological studies demonstrated that individuals with higher levels of toenail Se present a lower risk for type 2 diabetes [11]. However, the overexpression of selenoproteins and selenometabolites involved in carbohydrate metabolism at the supranutritional level was demonstrated to increase the risk for type 2 diabetes [12].

Brain function was reported to be affected both positively and negatively by selenium level. Selenium is not only an antioxidant for brain cells, but is also involved in neuronal signaling [13,14]. Decreased activities of selenoproteins determine neurological disorders and impaired cognitive functions [15]. Selenium and selenoprotein P protect the brain against Alzheimer’s disease [16,17]. However, selenium overexposure is neurotoxic, inducing lethargy and amyotrophic lateral sclerosis [18]. Inorganic forms of selenium are involved in the evolution of mild cognitive impairment in Alzheimer dementia [19].

**Figure 1:**
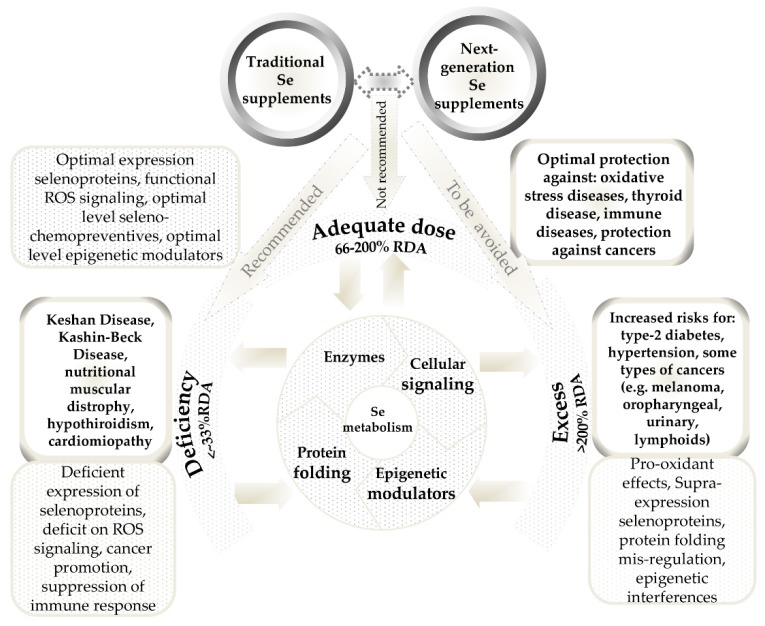
Physiological window of selenium supplementation. Intakes lower than recommended dietary allowance (RDA) determine diseases related to selenium deficiency. Intakes higher than 200% could lead to disorders related to excess selenium. The U-shape relationship between selenium and disease status limits selenium supplementation to subjects with lower-than-optimal selenium status. The next-generation selenium supplements, due to reduced toxicity and controlled release, should present a lower risk of supplementation on subjects with (near) Se optimal status. Data were obtained from References [5,23,43,44,45,46,47].

Selenium status is sub-optimal in various regions of the world—e.g., Europe and the Middle East [20], New Zealand [21], and northwest China [22]. Supplementation is recommended for people from selenium-poor regions [23]. Selenium is used as an ingredient in dietary supplements (DS), both in inorganic forms, such as selenite or selenate salts, mainly for multi-mineral and multivitamin supplements [24], as well as in organic forms, especially selenium-enriched yeast, as a stand-alone supplement [25]. However, the dual-side peculiarity of selenium, as an “essential poison”, is linked to a very narrow supplementation physiological window (Figure 1).

The recommended daily allowance (RDA) is considered to be 55 μg/day by the United States (US) Department of Agriculture [26]. A slightly higher and more specific RDA of 70 μg/day for men, 75 μg/day for lactating women, and 60 μg/day for women was recommended by the European Food Safety Authority (EFSA) [27]. The panel of experts from the US Institute of Medicine established the tolerable upper limit (UL) at 400 μg/day and the no observed adverse effect level (NOAEL) at 800 μg/day [28]. However, ingestion of 300 μg/day in a country with low–moderate selenium status determined the reduction of life expectancy [29]. In a selenium and vitamin E cancer prevention trial, a safe upper limit of ingestion of selenomethionine was considered as being 200 μg/day [30]. However, even this supranutritional level was considered to increase the risk for type 2 diabetes in susceptible persons [5].

Selenium implications for health are also related to its epigenetic effects [31], and as a redox regulator of genome, metabolome, and exposome [32]. The selenium complex action is exerted via bioactive selenoproteins and selenium metabolites. The selenoproteins are involved in many processes such as antioxidant activity, thyroid hormone metabolism, protein folding, redox signaling etc., but the mechanisms of action are not completely understood [33].

The selenometabolites modulate cell signaling, DNA methylation (directly or through one-carbon metabolism), histone acetylation, and finally, gene expression [31]. On mice, the use of metabolomic and transcriptomic techniques revealed that selenium supplementation involves a complex metabolic network, rather related to transcripts for fatty-acid β-oxidation and glucose transport rather than to selenoproteins [32]. Such epigenetics and redox-regulating effects could better explain the relationship between selenium supplementation efficacy and selenium base status [34,35] and/or genetic polymorphism [36] and suggest the need for a more personalized selenium supplementation approach. However, such a personalized selenium supplementation is not easy to implement, because assessment of selenium status is rather difficult and selenium supplements are freely available on the market, as nonprescription/over-the-counter medicine/dietary supplements.

Apart from the traditional Se formulations, a new generation of Se-based ingredients for dietary supplements is emerging (Figure 1). Such next-generation ingredients, represented mainly by zerovalent selenium nanoparticles [37] or selenized polysaccharides [38], slowly release the bioactive selenium species and have lower toxicity, more controlled and/or targeted mechanisms of actions, and fewer side effects. Despite the fact that such forms are relatively recently introduced and that they are not officially used in dietary supplements, the available information suggests that these already have a history of safe use. The widespread selenium yeast supplement was demonstrated to contain zerovalent selenium nanoparticles [39]. Biofortified mushroom, which is marketed as functional food, contains selenium polysaccharides [40]. Several specific features of these next-generation selenium ingredients, such as the slow release of active selenium forms, the ability to tailor different characteristics of selenium nanoparticles [41], or the synergistic antioxidant effects demonstrated by selenized polysaccharides [38,42], could make them more suitable for a personalized selenium dietary supplementation.

Selenium being an “essential poison”, necessitates fast, reliable, and affordable analytical methods, to be used by both the producers of dietary supplements and the controlling authorities for assessing and quantifying selenium. The proper analysis of each batch of Se dietary supplements is essential to guarantee benefits for human health. In some cases, acute intoxications from misformulated selenium dietary supplements were reported. The ingestion of an over-the-counter (OTC) tablet, which had 182-fold higher content than the labeled 150 μg of selenium, determined the occurrence of irritability, peripheral neuropathy, and fatigue in the US [48]. Similar neurological effects were reported after the consumption of another Se supplement, which was supposed to have contained an excessive amount of organic Se [49]. A more recent and detailed report was published on a misformulated dietary supplement containing 200-fold the intended dose of 200 μg [50]. The ingested dose exceeded 400 times the US RDA (55–70 μg/day). The symptoms and signs were also related mainly to neurophysiological disorders: memory loss and confusion, dizziness and imbalance, fatigue, irritability and anger, anxiety and depression, insomnia, headache, and eye and vision problems [51]. Due to Se supplementation’s narrow physiological windows, even smaller increases compared to labeled concentrations could have harmful effects.

In humans, Se plays an important role in preventing cellular damage induced by free radicals, by incorporation into antioxidant enzymes (selenoproteins) [52], and it has different biological roles depending on the specie, being essential for the brain [53], regulating thyroid function, and helping the immune system. It was also shown that selenoproteins can help reduce Hg toxicity in seafood [54]. A selenoprotein is any protein that includes a SeCys amino-acid residue. Examples of selenoproteins are five glutathione peroxidases (GPX) with antioxidant activity and three thioredoxin reductases (which, together with iodothyronine deiodinase, play an important role in regulating thyroid function; TrxR/TXNRD), and selenoprotein P, which is the most common selenoprotein found in the plasma. Proteins containing selenomethionine residues are not regarded as selenoproteins. Unspecific incorporation of SeCys instead of Cys in protein molecules could potentially cause proteotoxic stress, by inducing protein aggregation and inactivation [55,56]. The third most common amino acid, MeSeCys, which is non-proteinogenic, may have chemoprotective properties [57,58]. It is mainly found in Se-enriched plants from the genera *Brassica* and *Allium*, e.g., mustard greens, garlic, onion, and broccoli, as well as in Se hyperaccumulators from the genus *Astragalus* [59,60].

The complex selenium (bio)chemistry and physiology further complicate the selection of the analytical methods. Selenium species have different stabilities and biological activities. Inorganic selenium is a highly reactive prooxidant [61]. Volatile forms, such as hydrogen selenide and dimethylselenium, are generated from organic selenium [62]. Selenized yeast has a lower stability on the shelf—inappropriate storage conditions which promote selenium species interconversion [63,64]. Sodium chelate with amino acids/peptides, labeled as “selenium proteinate” or “selenium amino acids chelates” raised quality concerns [65]. The EFSA published a report regarding the inability of the expert panels to assess the safety of selenium amino-acid chelates [66]. In appropriate doses, selenized yeast was demonstrated to reduce lung, colon, and prostate cancer [67]. Selenomethionine was shown to have no effects on cancers in a trial organized by the (US) National Institutes of Health selenium and vitamin E cancer prevention trial (SELECT) [30]. In the case of next-generation dietary supplements, the analytical methods should differentiate the presence of the specific selenium ingredients, selenium nanoparticles (NPs), and/or selenized polysaccharides.

The aim of this study was to review the existing analytical methods for selenium quantification and speciation from dietary supplements and to evaluate the need for further developments in terms of extraction and separation, especially required by the emerging next generation of Se dietary supplements. Our analysis was done in comparison with the methods used for the Se dietary supplements already existing in the market.

## 2. Conventional and Next-Generation Selenium Dietary Supplements

Selenium (Se) is an important chemical element due to the speciation forms it presents [18]. In the environment, Se presents five oxidation states: +4, +6, 0, −1, and −2, known under the following forms: selenite (Se^+4^, SeO_3_^2−^, HSeO_3_^−^), selenate (Se^+6^, SeO_4_^2−^), elemental selenium (Se^0^), selenide (Se^2−^), and organic selenium (mainly selenomethionine (SeMet), selenocysteine (SeCys), and methylselenocysteine (MeSeCys)) [68,69,70].

The most common species of selenium in traditional dietary supplements are inorganic selenium (iSe), especially sodium selenite (Se^+4^), but also selenate (Se^+6^), selenide (Se^−2^), and Se^0^; and organic Se, in SeMet, SeCys, MeSeCys, and selenized yeast (Se-yeast) rich in SeMet. Some reports found other types of selenium species, such as phenylselenocysteine, methaneseleninic acid (methylseleninic acid, MSeA), and selenocyanate to be also relatively common [71]; however, apparently, there are no known supplements labeled as containing these ingredients [72]. SeMet and SeCys are the two amino acids under which Se is found in proteins, SeCys being known for its reactivity to form Se–Se and S–Se bridges [73].

**Figure 2:**
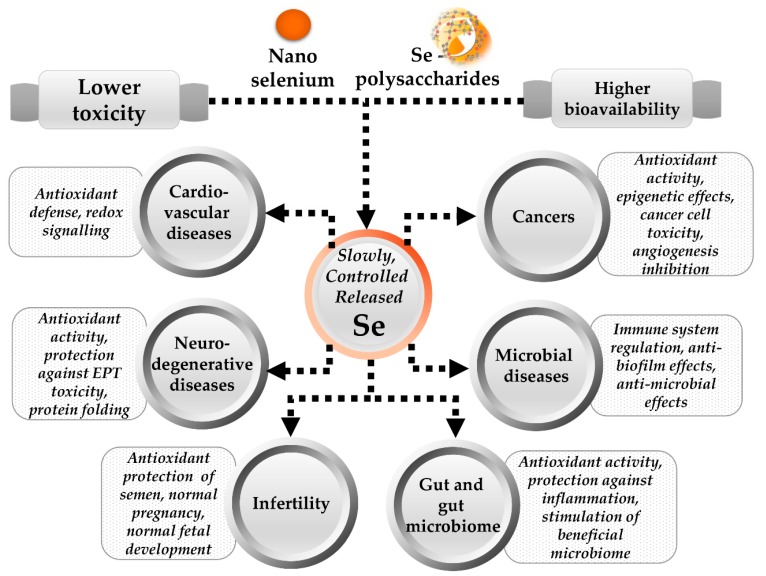
Potential benefits of the next-generation dietary supplements based on nanoselenium and selenium polysaccharides. The new forms of selenium ingredients were proven, in several experimental models, to have lower toxicity and higher bioavailability. The biological activities of such putative slow, controlled-release selenium forms are not limited to the already known effects of selenium. Additional effects, such as in vitro inhibition of biofilm formation by the multi-antibiotic resistant pathogenic bacteria or stimulation of beneficial microbiome on chicken, were recently reported for these new forms of selenium. Data were obtained from References [14,33,37,41,74,75,76,77,78,79].

Next-generation selenium supplements, with potential lower risk for excess supplementation, are emerging (Figure 2). These are based on selenium forms with lower toxicity, higher bioavailability, and presumptive controlled release, such as selenium zerovalent nanoparticles (SeNPs) and selenium polysaccharides (SPs). The new ingredients, SeNPs and SPs, should present a lower risk for excess Se supplementation on subjects with optimal status, due to putative slow and consumption-controlled release of bioactive species.

The bioavailability and biological activity of selenium depend not only on the total ingested amount, but also on its chemical form, solubility, digestibility and accessibility, the presence of other dietary components, and the physiological status of the organism [25,80,81]. The organic forms are less toxic and are absorbed more effectively (especially SeMet) than the inorganic ones [82,83]. Inorganic Se (Se^+4^ and Se^+6^) is 40 times more toxic than organic Se [84], and Se^+4^ is nearly 10 times more toxic than Se^+6^ according to the US Environmental Protection Agency (EPA) report (FRL–5649–7) [85]. In terms of human nutrition, organic compounds have a higher bioavailability and are assimilated from food/supplements in ranges of 85%–95%, while the uptake range for inorganic selenium is 40%–50% [86].

The most abundant specie in Se-yeast was found to be SeMet, but there are over 60 types of Se species reported [24]. The common method of characterizing and comparing Se-yeast was by quantifying the total SeMet content, and the speciation analysis traditionally involves the determination of low-molecular-mass selenospecies. This approach has the disadvantage that it does not properly quantify the bioavailability of Se, as SeMet can be both free and protein-bound, and only the free form is available for replacing methionine in proteins, via non-specific incorporation. Enzymatic digestion usually used to liberate SeMet from proteins for quantification in Se-yeast is a powerful tool, but presents several difficulties, such as differences in SeMet release.

Recently, Fagan et al. assessed the free, peptide-bound, and total water-soluble SeMet in four commercially available Se-yeast products, and showed that there are significant differences between Se-yeast products, as well as in comparison to the certified reference material, SELM-1 (selenized yeast reference material, produced by the National Research Council of Canada, Institute for National Measurement Standards). The differences imply that the preparations also differ in properties such as bioavailability, bio-efficacy, shelf-life, toxicology, etc. Based on LC–MS/MS and quantitative proteomics, they identified over 62 Se-containing proteins [25]. Torula yeast (*Candida utilis*) seems to metabolize Se in a specific way, different from Brewer’s yeast (*Saccharomyces cerevisiae*), where the most abundant compound is selenohomolanthionine (SeHLan) [87].

An alternative to Se-yeast suitable to be used as natural Se-rich ingredients in dietary supplements could be represented by Se-enriched mushrooms (macro-fungi) such as *Pleurotus* sp. [40,88,89] and *Agaricus* sp. [90]. Mushrooms are known as good bioaccumulators of minerals and other elements [91], including selenium, even when grown in soils with low content [92]. The Se content in several mushrooms was reported in the past decade, with values ranging from sub-μg to hundreds of μg, depending on the mushroom species and the growth conditions, such as Se concentration and substrate used [89,90]. In general, the bioaccumulation of Se in biofortified mushrooms was reported to be proportional to the concentration of supplemented Se, as long as this concentration did not become toxic [89].

Furthermore, probiotic bacteria capable of accumulating selenium were investigated as potential supplements. In selenium-enriched lactic acid bacteria *Enterococcus durans* LAB18s, Se appears to accumulate mostly in the protein fraction (particularly alkali-soluble), followed by polysaccharides and nucleic acids [93]. Other Se-enriched probiotic bacteria such as *Lactobacillus* spp. also started gaining a lot of attention lately, especially for dietary supplementation in animal feed, but also for preliminary testing of their biological effects [94,95,96,97].

Another option for compensating Se deficiencies is to use selenium-rich vegetables and sprouts grown in special conditions, which were found to have better chemoprotective effects than Se^+4^ species and SeMet. A study carried out with six types of sprouts, most of them from the family *Brassicaceae*, showed that the seedlings germinated and grown in the presence of sodium selenite, but not sodium selenate, could convert inorganic selenium into MeSeCys and SeMet [59].

The use of elemental Se in the form of NPs for different biological applications gained a lot of attention lately, because of several proposed advantages compared with other forms of Se, including higher bioavailability [98], lower toxicity [41,99,100,101], potential for local delivery of high doses into cancer cells [102]; possibility to be modified, stabilized and functionalized by various polymers such as chitosan [103,104,105,106] or other polysaccharides [107,108,109,110,111,112], proteins [113,114], or even combinations between them [115,116,117], smaller molecules such as polyphenols [118,119,120,121], sialic acids [122,123], folic acid or folate (as folate–chitosan smart-shell nanocapsules) [124,125,126], and other organic acids [37]. Chemical, physical, or biological methods are used for producing SeNPs [37], each having its own advantages and disadvantages. Depending on the type of method used to synthesize SeNPs, their morphology, size, composition, and properties can be different. One of the important aspects of SeNPs is represented by the presence or absence and the nature of the stabilizing coating molecules, such as proteins, polysaccharides, or even smaller molecules, such as polyphenols. These characteristics influence the bioavailability, toxicity, and effects of NPs by influencing the size, surface electric charge, and hydrophobicity of NPs. In the case of using SeNPs in dietary supplements, the studies are still at an early stage.

One possible, but little studied form of selenium supplementation is via complexation with humic/fulvic acids. Humic acids (HA) and HA-like biopolymers were proposed to have several beneficial effects on human health, such as positive impacts on the colonic microbiome [127] and on the immune system [128], antiviral activity, anti-inflammatory effects, protection against ionizing radiation, etc. [129]; however, they can also have negative effects, such as cytotoxicity toward vascular endothelial cells [130], increased oxidative stress of red blood cells [131], and growth retardation and apoptosis of fibroblasts, which were proposed to explain its possible role as an etiological factor of Blackfoot disease in Taiwan [132]. Humic/fulvic acids and selenium salts were previously shown to reduce each other’s toxic effects in mice and rats [133]. A recent study showed that the biogenic SeNPs stabilized by humic-like substances from the extracellular polymeric substances (EPS) produced by anaerobic granular sludge biofilms had decreased bioavailability and toxicity on zebrafish embryos, in comparison to chemogenic SeNPs and selenite [134]. Several investigations of SeNPs toxicity toward aquatic and other organisms showed that the concentration, the form and characteristics, as well as the presence of other compounds, influence the degree of toxicity [100,135,136,137,138]. There is a scientific opinion published by the EFSA journal on the data provided by a petitioner, in which it was stated that there were insufficient data to conclude on the safety and bioavailability of selenium humic/fulvic acid chelates [139]. To the best of our knowledge, there is no other information with respect to this type of selenium supplement, but the subject deserves further attention.

One relatively ignored aspect is that Se-yeast, such as selenized *Saccharomyces cerevisiae*, which is extensively used in nutritional supplements [25], as well as probiotic bacteria and possibly mushrooms, also contain SeNPs, because they are able to form them as a way of reducing Se toxicity [93,140]. Moreover, SeNPs can be released into the extracellular space in a time-dependent manner. The possibility that this release also takes place inside the intestines, as well as the possible implications, should be investigated. For SeNPs to be safely used in supplements, processes such as absorption and metabolism in the gastrointestinal tract (GIT) need to be addressed. The mechanisms were previously reviewed [37,41,141,142,143] and are not going to be described in detail here. It is important to mention that the study of NPs’ fate after oral exposure is complicated by the complexity of the biological system which could lead to in vivo surface modifications. There is not much information available with respect to the transformation induced on NPs in the gastrointestinal tract upon oral administration. Transport, absorption, translocation, excretion, and other processes are influenced by several parameters, such as NP size and its dependence on pH (including the pH in the GIT) and other factors, NP surface chemistry and properties, and interactions with different biomolecules, especially proteins [37]. Moreover, some reports also suggest that at least some of the biological activities are size-dependent, with a smaller size of NPs inducing higher activity [144,145,146], although this process could be less pronounced at a high level of Se deficiency in cells [145]. Therefore, it is important to predict and control these processes.

Another next-generation group of selenium supplements was proposed under the form of SPs, which are extra- and intracellular polysaccharides (EPS and IPS, respectively) modified with selenium. They were mainly investigated for their strong antioxidant properties [147,148,149,150,151,152]. Many selenized polysaccharides were obtained via physico-chemical methods, involving heating or microwaving polysaccharides purified from different biological sources, in H_2_SeO_3_/HNO_3_ with Ba^2+^ as a catalyst [149,150,153]. For SPs to be used in dietary supplements, they should be ideally obtained via more biocompatible ways, such as green extraction methods of selenized polysaccharides that occur naturally in different food sources and that can even be enriched by soil supplementation with selenium. These methods were already investigated by several groups [38,42,147,148,151,152,154]. Unfortunately, until now, these methods prove to be time consuming and less efficient in terms of yield compared with the chemical methods, although more and more studies are focusing on optimizing them [42,151,152,155].

Selenized polysaccharides from mushrooms, green tea, and other biological sources were investigated as potential protective factors against lipopolysaccharide (LPS)-induced acute kidney injuring (AKI) and its complications [38], H_2_O_2_-induced cytotoxicity [152], proliferative activity of several cancer types [153], oxidant activity [152], diabetes [156], etc. The available information suggests that Se content in selenized polysaccharides is one of the most important factors influencing the anti-proliferative and anti-tumor effects, although other factors such as substitution patterns, molecular structure, monosaccharide composition, average molecular mass, water solubility, etc. may also play a role [153,157,158]. Microbial and plant polysaccharides were already shown to possess important biological activities that are influenced by these parameters [157,158,159,160]. The subject still needs further investigation in order to elucidate the relationship between structural features and selenium content, on one hand, and activity, on the other hand.

One aspect which is not sufficiently explored in the literature is the fact that the concentration of selenium has an influence on its biotransformation route in microorganisms. This is due to its dual nature, i.e., an essential and toxic trace element at low and high concentrations, respectively. The microorganisms can incorporate Se into their biological molecules, such as proteins and polysaccharides, proportional with available Se concentration, as long as this concentration does not become toxic, in which case they will start reducing the inorganic specie to Se(0) and form NPs [161,162]. This, together with the fact that the toxicity inhibits growth and metabolism, and that Se is incorporated mainly in proteins [161], could be some of the reasons for low selenized polysaccharides yields. In order to get only organic incorporation, such as selenized polysaccharides, either the concentration of inorganic selenium needs to be kept at an optimal value or the organic fraction needs to be purified from NPs, which increases the production cost. In the case of polysaccharides, the yield would be, nevertheless, low in both cases.

Table 1 presents several biological positive effects of the two most investigated forms of Se with high potential to be used in dietary supplements, the method of synthesis, and the experimental biological model.

**Figure 3:**
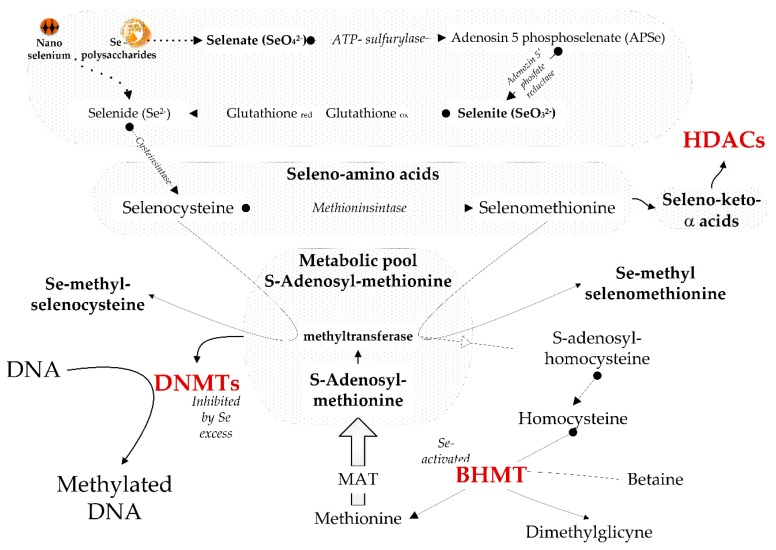
Next-generation Se supplements and their (potential) moderate epigenetic effects. In red are enzymes influenced by Se level. A low level of Se disturbs one-carbon metabolism and *S*-adenosyl methionine restoration, because betaine homocysteine methyltransferase (BMHT) needs higher levels of selenium for optimal activity. A high level of Se leads to inhibition of DNA methyltransferases (DNMTs) and to competition for methyl groups, needed to produce methylated selenium amino acids. Excess selenium influences histone deacetylation and further cross-talk with DNA methylation. The seleno-amino acids are converted via transamination to seleno-keto-α-acids, which inhibit histone deacetylase (HDACc). Zerovalent selenium and selenium polysaccharides slowly release active selenium species via an equilibrium reaction. The rate of such an equilibrium reaction depends on consumption of the reaction products. In tissues of subjects with a normal/optimal selenium status, the consumption of the reaction products is lower and the release of active selenium species over the optimal status is slower. At a deficit-to-optimal level of intake, the selenium species are rapidly used for the expression of the deficit amino acids and seleno-proteins, and the rate of release is higher. The slow and consumption-controlled release of the bioactive Se species should allow a more adapted response of supplementation to selenium status. Figure modified from Oancea et al. [186]. Data were obtained from References [31,37,186,187].

As already mentioned, Se also exerts epigenetic effects, either directly, via inhibition of DNA methyltransferases (DNMTs) and histone deacetylase (HDACc), or indirectly, via activation of betaine homocysteine methyltransferase (BMHT), a key enzyme for one-carbon metabolism and *S*-adenosyl methionine restoration. The next generation of Se supplements might have more equilibrated epigenetic effects compared to traditional forms, due to the slow and consumption-controlled release (Figure 3).

## 3. Analytical Methods for Selenium Detection and Speciation

### 3.1. Total Selenium

The chemical element of Se has the atomic number 34. The most abundant selenium isotope is ^80^Se (49.61%), followed by ^78^Se (23.77%). There are also less abundant isotopes, such as ^76^Se (9.37%), ^82^Se (8.73%), ^77^Se (7.63%), and ^74^Se (0.89%). This isotope distribution generates a profile that allows Se differentiation from other chemical elements [188].

The newest/most recently developed method for total Se determination is inductively coupled plasma mass spectrometry (ICP-MS). This is done on samples submitted to nitric acid digestion (HNO_3_), usually assisted by hydrogen peroxide (H_2_O_2_). ICP-MS is used both for the determination of total (inorganic) selenium from dietary supplements and for the speciation of Se^+4^ and Se^+6^. The other two most used methods are atomic absorption spectroscopy (AAS) and, to a lesser extent, inductively coupled plasma optical emission spectrometry (ICP-OES). All methods can be and are usually coupled with hydride generation (HG), via techniques being known as HG-ICP-MS, HG-AAS, and HG-ICP-OES, respectively, in order to transform inorganic selenium into volatile hydride H_2_Se. HG also eliminates the risk of volatilization and loss of the analyte of interest due to the use of mineral acids for the initial digestion of (organic) samples. Another possibility to eliminate this risk is the use of the laser ablation ICP-MS (LA-ICP-MS), which can analyze Se from solid samples, in order to avoid the acid digestion that is usually employed before analysis.

The HG method implies derivatization of the sample with sodium tetrahydroborate (III) which allows the quantification of the hydride-forming elements, selenium belonging to this category, according to one study [189]. However, Se^+6^ should be reduced to Se^+4^ beforehand, because it does not react with sodium borohydride and HCl to form selenium hydride [190,191]. In addition to increased sensitivity, the hydride generator separates As and Se from chloride interferences, improving the detection of these elements in matrices such as sea water [192].

The AAS methods usually used for Se are graphite furnace AAS (GF-AAS) and electrothermal AAS (ET-AAS). Flame AAS (FAAS) is not suitable and is not much used, because of the poor emission characteristics of Se. High-resolution continuous source (HR-CS) coupled with GF-AAS was used as a derivative method for determining Se from multivitamin dietary supplements [193].

Each of these methods has advantages and disadvantages. ICP-MS suffers from interferences from plasma gas or other elements present in the sample, including ^36^Ar^40^Ar, ^38^Ar^38^Ar, ^38^Ar^40^Ar, ^40^Ar_2_, ^79^Br^1^H, and ^40^Ar^37^Cl [194], which can be managed by using collision or reaction cells. Another option is to use the isotope ^82^Se for quantification, based on its known prevalence in nature [195] or ^78^Se in samples with high bromide concentration [196]. The set-up and operational costs are usually much higher for ICP-MS than for other elemental analysis techniques, and it needs specialized and qualified personnel. The advantages of ICP-MS include the fact that it can handle both simple and complex matrices with less matrix interference due to the high temperature of the ICP source and it presents a detection limit (ppt range) and sample throughput for most elements better than those obtained using AAS or ICP-OES [197]. It detects the largest number of elements, and some isotopic information can be obtained with this method. Additionally, a small sample volume is required. Another study demonstrated the advantage of using ICP-TOF-MS in dietary supplement (DS) analysis with a complex and variable matrix by simultaneously mapping the elemental composition with very good sensitivity and accuracy [198].

The major disadvantages of HG-AAS are the interference of transition metals such as Mn, Zn, and especially Cu, which affect the formation of H_2_Se, the use of concentrated acids to destroy the organic matter before analysis, which sometimes also gives incomplete mineralization, and the need of a large sample size. It has the advantage that it can selectively separate Se from the matrix by generating volatile covalent hydrides, thereby allowing minimum matrix interferences.

GF-AAS offers the possibility of determining selenium directly from food supplements, with minimal risk of loss of sample and contamination. This technique also has the following advantages: high sensitivity, low consumption of toxic reagents, and reduced analysis time. High-resolution continuous source (HR-CS) coupled with GF-AAS is another alternative for determining Se from multivitamin dietary supplements [193]. Electrothermal atomic absorption spectrometry (ET-AAS) [199], previously used for the quantification of Se from medicinal herb aqueous extract, is a very sensitive technique with good accuracy, being useful especially for the determination of very small amounts of Se, having a limit of detection (LOD) <5 mg·L^−1^. Both GF-AAS and ET-AAS need a matrix modifier in the case of organic samples, because they are sensitive to matrix interferences [200,201,202]. Additionally, strong oxidizing conditions (usually nitric acid + hydrogen peroxide) are needed in the case of ET-AAS, to prevent the volatilization of Se [200,203].

HG-ICP-OES has the same disadvantage and advantage as HG-AAS, of requiring concentrated acids and minimum matrix interferences, respectively. Additionally, it has a higher detection limit compared with other techniques, especially ICP-MS, due to the poor emission intensity of Se. It is not used as much as the other techniques. Both ICP-based methods (ICP-OES and ICP-MS) suffer from two interferences related to changes in plasma properties and in sample uptake rate in the nebulizer. These can be compensated for by using appropriate standards.

Another technique, photochemical vapor generation (photo-CVG), offers several advantages when applied to analytical atomic spectrometry for selenium determination, such as reducing interferences and allowing speciation via conversion under ultraviolet (UV) irradiation into volatile fractions [204]. Recent research demonstrated that electrospray ionization (ESI)-MS can be used as a selenium-specific detector, free of polyatomic interferences that affect ICP-MS [73].

Total reflection X-ray fluorescence spectroscopy (TXRF) is a technique able to detect selenium at the ppm level, with a detection limit of 0.1 to 0.2 mg/kg being reported for dietary supplements. TXRF has the advantage of operation simplicity, requiring minimum preparation of the samples. However, its sensibility is low and its operation requires a special work permit and high-energy exposure precautions [205].

One relevant field in analytical chemistry for the analysis of selenium supplements is the speciation of elements [206], which allows one to determine the forms under which Se is present. Given the importance and role of selenium in the human body and in dietary supplements, it is very important to develop accurate analytical methods to determine existing selenium forms and their properties. Total Se and Se speciation should be determined in a complementary manner.

### 3.2. Inorganic Selenium

Selenium commonly exists in compounds in the oxidation states −2, +2, +4, and +6. Selenous acid salts are called selenites—e.g., silver selenite (Ag_2_SeO_3_) or sodium selenite (Na_2_SeO_3_). Salts of selenic acid are called selenates (SeO_4_^2−^). Selenium forms hydrogen selenide, H_2_Se, which is a highly toxic, colorless gas, with a strong smell. Moreover, reduction of Se to H_2_Se by thiols such as gluthatione was shown to be letal to *Saccharomyces cerevisiae* in micromolar doses, compared to millimolar doses for selenite [207].

Selenite is the dominant selenium species in aquatic habitats. Selenite was found to be more toxic than selenate [208,209,210], and is, therefore, the most investigated form of selenium species, although the opposite order of toxicity was also reported [211]. Optical detection techniques include colorimetry, absorbance, and photoluminescence, but their accuracy depends on the characteristics of the sensory material. Inorganic selenium forms can be detected in a water matrix using voltametric methods. The voltametric and optical methods have the potential to lead to the development of inexpensive portable devices for inorganic selenium detection in water [212].

Selenite concentrations can be quantified using hydride generation atomic absorption spectrometry (HG-AAS) [213,214]. Individual species concentrations of Se^+4^ and Se^+6^ can be determined using different techniques; however, Se^+6^ is converted to Se^+4^ and there are possible oxidation/reduction reactions during sample extraction [72]. As of 2012, high-performance liquid chromatography in conjunction with hydride generation atomic absorption spectrometry detection (HPLC–HG-AAS) is used for determination of Se^+4^ and Se^+6^. However, the method needs to be validated using certified reference materials for the determination of inorganic selenium species [215].

For the determination of Se^+4^ and Se^+6^, a UV photochemical vapor generation system (UV-PVG) coupled with AAS was developed, using formic acid as the photochemical agent for UV transformation of Se^+4^. The advantage of this method is the very low limit of detection (LOD = 40 ng·L^−1^) and low reagent consumption [216].

A simple and selective method for determination of Se^+4^ uses the complexing reagent 4-(4′-chlorobenzylideneimino)-3-methyl-5-mercapto-1,2,4-triazole (CBIMMT) in dichloromethane, which forms an orange-colored complex with Se^+4^ The concentration is then measured using spectrophotometric methods (λ_max_ = 470 nm) [217].

### 3.3. Organic Selenium

Organic Se in different samples may be calculated by subtracting inorganic Se from total Se; however, for speciation, a more detailed analysis is required. Except for supplements that are specifically based on SeMet, other common products, like Se-enriched yeast or mushrooms, include a multitude of complex molecules in which selenium is incorporated. In order to assess their bioavailability, such products should be digested in conditions similar to those in the human body, and then subjected to detection methods, such as HPLC combined with other techniques. Thus, chromatographic separation can be followed by mass spectrometry in order to identify interactions between Se and Hg [194]. A study on selenium metabolic compounds in plants grown in medium enriched with inorganic selenium led to the identification of MeSeCys, SeMet, γ-glutamyl-MeSeCys and inorganic SeO_3_^2−^ using HPLC–ICP-MS. The method also detected some unknown compounds containing selenium the [195]. Analytical methods based on GC can be used to detect especially volatile Se species such as (CH_3_)_3_Se^+^, (CH_3_)_2_Se, and CH_3_SeH [218].

The UV-ICP-MS detection system with sequential online exclusion chromatography was used to quantify different fractions with a low detection limit. The instrumental coupling adopted offered the advantage of a selective and sensitive method [219].

Recently, Kubachka et al. analyzed selenium as selenate, selenite, selenium yeast, MeSeCys, SeMet, selenium amino-acid complexes, or chelates, from 13 dietary supplements in various forms, e.g., capsules, soft or liquid gels, and tablets, using LC–ICP-MS, which offers the advantage of separation of analytes of interest prior to their quantification via ICP-MS. This study developed several extraction methods (0.1 M NaOH, 4 M methane sulfonic acid (MSA), water, and 1 M HCl) for the determination of inorganic selenite forms Se^+4,+6^ and organic MeSeMet (which represents unretained Se species), MeSeCys, MseA, MeSeOCys (represented by SeCys_2_), SeMet, and SeOMet. Based on the obtained results, a discrepancy between labeled ingredients and the analyzed species was observed [72].

Among minor selenium organic compounds, selenocystine (SeCys_2_) is constantly reported in various samples—e.g., it was reported to exceed Se-methylselenocysteine (Se-MeSeCys) in dicotyledoneous plants [220]. However, in the above cited papers of Kubachka et al. [72], it was demonstrated that the peak identified as SeCys_2_ via retention time on HPLC was, in reality, Se-methylselenocysteine oxide (MeSeOCys).

The four major types of analytical methodologies to quantify and/or identify proteins such as Gpx1 include enzymatic assay, polyacrylamide gel electrophoresis (PAGE) with Western blot detection of proteins, or with inductively coupled plasma mass spectrometry (ICP-MS) detection of selenium, or with proteomics based on LC–MS/MS, and size-exclusion chromatography with ICP-MS detection. Better sensitivity is obtained with enzymatic assays and immunodetection, but the drawbacks include limited selectivity and limited dynamic range, which compromise accuracy [221].

A large number of dietary supplements are currently based on Se-enriched yeast (*Saccharomyces cerevisiae*), and the speciation analyses of such products revealed, on one hand, the presence of a large number of Se metabolites with an uncertain impact on human health and, on the other hand, large differences in analysis results, depending on the methods that were used for proteolysis, separation, quantification, etc. Therefore, it is necessary to develop more reliable Se speciation protocols, in order to monitor the fermentation processes and direct them toward beneficial compounds, and also to ensure consistent product quality. The two main amino acids found in yeast selenoproteins are SeMet and SeCys. The quantification of SeMet is well established, but it was found to depend on granulometry and other characteristics of supplements. There are still challenges related to SeCys quantification, because it is unstable; thus, it should be derivatized after hydrolysis (e.g., via carboxymethylation). The analytical methods used for Se speciation include HPLC followed by time-of-flight MS, Orbitrap MS, and Fourier-transform ion cyclotron resonance mass spectrometry. Each method has advantages, disadvantages, and limitations, and the most recent approach is to combine, for instance, HPLC with both electrospray ionization (ESI)-MS and ICP-MS. Although ICP-MS is robust and accurate, ESI–MS is able to identify peaks that are missed by ICP-MS. ESI-MS is sensitive to the presence of salts; thus, the results depend upon the purity of the sample [56]. Using a complex combination of one-dimensional (1D) isoelectric focusing electrophoresis (IEF), 1D SDS-PAGE, 2D IEF PAGE with LA–ICP-MS, and capillary HPLC–ICP-MS/electrospray Orbital MS/MS, Bianga et al. managed to identify and semiquantify the proteins (glutenins and gamma-gliadin) that incorporated selenium in wheat; moreover, they managed to detect not only unspecific selenomethionine, but also selenocysteine incorporation [222]. In a recent protocol from Methods in Molecular Biology, other recent cases of applying LA–ICP-MS imaging together with other methods, such as the ones mentioned above, for Se detection and mapping not only in proteins, but also in biological (animal or plant) tissues were reviewed [223].

In addition to these techniques, graphite furnace atomic absorbtion spectroscopy (GF-AAS) was used for Se analysis and enzymatic digestion HPLC for speciation in Se-enriched sprouts [59], ICP-OES was used to quantify organic forms in *E. durans* [93], and a method based on hydrophilic ion interaction chromatohraphy (HILIC) with ICP-MS, HILIC–Orbitrap MS, and MS/MS fragmentation was recently developed to identify SeHLan in Torula yeast [87].

Liquid chromatography (LC) in combination with HG atomic fluorescence spectrosocopy (HG-AFS) was used for selective analysis of selenite, selenate, selenocystine, and SeMet [224], and high-performance liquid chromatography with hydride generation atomic absorption spectrometry (HPLC–HG-AAS) was used to analyze the species of Se present in DS. Another technique used for DS analysis is microwave plasma atomic emission spectrometry (MP-AES), with low operating costs, which does not require flammable gases and can be an alternative to flame atomic absorption spectrometry (FAAS) in terms of selectivity. For analysis of Se^+4^ and SeMet in biofortified yeast, ion-pair reversed phase HPLC–HG-MP-AES was used, being a relatively simple method with a quantification limit for Se^+4^ of 11.9 mg·g^−1^ and for SeMet of 104 mg·g^−1^ [225].

Analyzing the previously reported data on the selenium content of mushrooms, a relatively recent review draws attention to the biased or imprecise data sometimes reported in the literature for biological samples, due to improper methods used for detection [226]. The review highlighted once more the drawbacks of conventional configurations of techniques such as classical ICP-OES, classical flame AAS, or ICP-MS, and the advantage of using hydride generation. Other techniques such as fluorimetry, instrumental neutron activation analysis (INAA), and GC were appreciated as methods that usually give accurate results.

### 3.4. Selenium Next-Generation Ingredients

The two main Se species with high potential to be used as ingredients in the next-generation Se supplements are selenized polysaccharides and SeNPs.

In the case of SPs, the molecular weight can be determined with high-performance gel permeation (or gel filtration) chromatography [149,156,227,228,229], and the presence and quantification of Se can be confirmed by ICP-OES, ICP-MC, or AAS [149,182,229]. More in-depth characterization can be obtained with Fourier-transform infrared spectroscopy (FTIR), UV spectroscopy, and AFM [156]. Moreover, SPs can be hydrolyzed and the monosaccharides constituents identified. For example, Liu et al. isolated a selenium polysaccharide from *Catathelasma ventricosum* mycelia with glucose as the most abundant monosaccharide and much lower amounts of mannose, xylose, and galactose [156]. The atomic force microscope used revealed the branched structure of the SPs. Additionally, they confirmed the absence of uronic acids using high-performance anion-exchange chromatography.

In the case of selenium nanoparticles (including those within cell biomass samples), X-ray fluorescence analysis (XFA) and electron energy-loss spectroscopy (EELS) can be used to detect the presence of Se, and Se oxidation state can be determined with X-ray absorption techniques (X-ray absorption near-edge structure (XANES) and extended X-ray absorption fine structure (EXAFS)) [230,231].

For morphological and structural characterization, techniques such as atomic force microscopy (AFM), scanning electron microscopy (SEM), transmission electron microscopy (TEM), or X-ray diffraction (XRD) are used [105,232,233,234,235]. Energy dispersion X-ray spectroscopy can be also used to identify Se [236] and it can be associated with TEM or SEM. Another important parameter is the size of NPs, which is usually determined with dynamic light scattering (DLS), gel permeation/size exclusion chromatography (SEC) or TEM. Other methods such as UV–Visible (UV–Vis) and vibrational (mainly FTIR and Raman) spectroscopy are also used for characterization.

These techniques are too expensive and cumbersome to be implemented as a routine in analysis of dietary supplements and do not provide all the necessary information individually. Generally, these methods give mostly qualitative information with respect to NP detection, aspect, size, and/or morphology, and less quantitative information. Another major drawback of these methods is their difficulty in detecting, characterizing, and quantifying SeNPs at low concentrations (μg·kg^−1^).

Therefore, it is necessary to develop new analytical and physico-chemical methods to determine and characterize SeNPs in dietary supplements, but also to predict their transformation and activity upon ingestion. Several alternative methods were already developed or classical methods for selenium were adapted to NPs. Recently, alternatives to TEM, such as (a)symmetrical flow field-flow fractionation (FFF), coupled with different detectors [237,238], or single-particle inductively coupled plasma mass spectrometry (SP-ICP-MS) [39], were proposed for detecting and characterizing SeNPs. Asymmetrical flow field-flow fractionation (AF4) coupled with a matrix diode array detector (DAD) and ICP-MS (AF4–UV-ICP-MS) was developed and applied for characterization and size determination of SeNPs synthesized by reducing selenite with ascorbic acid in the presence of stabilizers such as polysaccharides (chitosan (poly (d-glucosamine) and hydroxyethylcellulose (HEC)). The NPs were detected with three orders of magnitude smaller than other detection systems such as dynamic light scattering (DLS) or multiple-angle light scattering (MALS), allowing unequivocal NP identification. UV–Vis spectrometry was used as an AF4 detector, in order to select the best conditions of separation in a fast and inexpensive manner compared to the use of ICP-MS [232]. Moreover, based on a combination of online FFF with ICP-MS and zeta potential, one group investigated the size, concentration, and charge of SeNPs stabilized by different biomolecules such as polysaccharides or proteins, under GIT relevant conditions (pH and enzymes). FFF offers the advantage of a relatively gentle separation process and also allows the fractional samples to be collected for further analysis using other techniques, in this case, ICP-MS quantification. The use of different types of stabilizing molecules leads to different NP sizes which could be a result of differences in steric repulsion, electrostatic interactions, and/or conformation/structure of biomolecules. The influence of pH on NP size very much depends on these parameters, but an important aspect is that more than 90% of Se retained the nanoparticle form, as determined by online flow-field fractionation coupled with ICP-MS [231]. The major advantage of the technique is the ability of continuous, non-destructive separation, with a high resolution between 1 and 100 nm, which allows it to be applied for the separation of very small particles [239].

**Figure 4:**
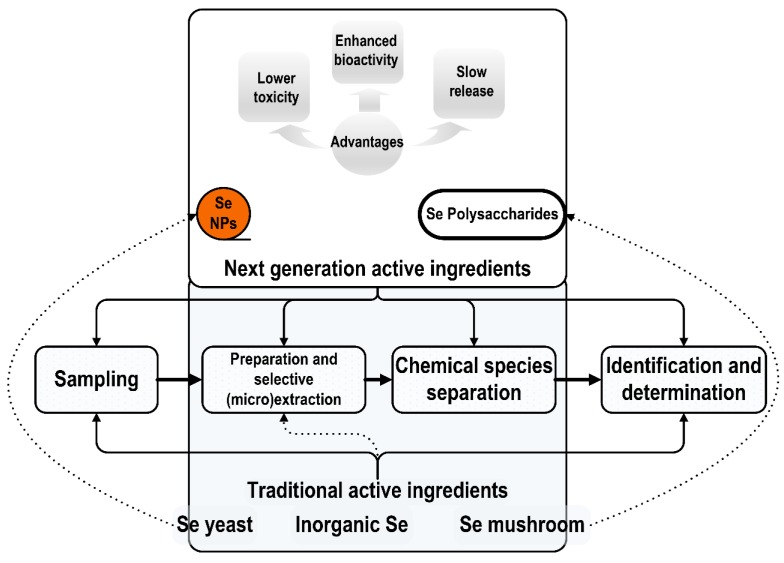
Schematic representation of the analytical procedures for determining and characterizing selenium in dietary supplements. The analysis of the next-generation selenium dietary supplements based on nanoselenium or on Se polysaccharides must include a step related to chemical species separation. Such a step would allow a proper characterization of the molecular mass/dimension of Se-based ingredients and it is mandatory to substantiate the marketing claims related to the main advantages of these new Se ingredients—lower toxicity, enhanced bioactivity, and slow and controlled release.

The methods of analysis for the next-generation selenium supplements should include a step related to chemical species separation. Such a step would allow a proper characterization of Se forms/species, including molecular mass/dimension, and substantiates the marketing claims related to the main advantages of these new selenium ingredients (Figure 4).

In the case of SP-ICP-MS, a very diluted suspension (aqueous solution) is introduced into the ICP-MS instrument such that, from a statistical point of view, only one nanoparticle enters the plasma at a certain moment. The plasma atomizes and ionizes the nanoparticle constituents. These nanoparticle constituents are then quantified using the mass spectrometer. The parameters of the nanoparticle population which can be measured include nanoparticle mass concentration, nanoparticle concentration, average size, and size distribution. A unique advantage of this technique is represented by its very high detection capacity of nanoparticle number or mass concentration. Thus, the technique could be used to detect and to characterize nanoparticles which are present in environment samples at extremely low concentrations [240].

However, there are also some disadvantages of this technique [241], as follows:

- In a single analysis using quadrupole instruments, which is the most common type of an ICP-MS instrument, it can measure only one isotope or maximum two isotopes;
- The nanoparticle size detection limit (LOD), expressed as the equivalent spherical diameter, ranges from 10 nm to 20 nm for monoisotopic nanoparticles.

For NPs of elemental selenium, a detection limit of 200 nm was theoretically estimated [242] calculated using a low-abundance isotope ^76^Se (9.36%). This limit can be improved using reaction/collision cells [243] or using mathematical correction equations [244]. It requires a stage of real sample preparation to be compatible with the common sampling systems in ICP-MS, which is also a valid requirement for other techniques [241].

An analytical method based on SEC and SP-ICP-MS was developed for the detection and size characterization of SeNPs in yeast, in order to include this form in the speciation scheme of Se-yeast [39]. To significantly reduce the detection limits for SeNP characterization in Se-rich yeast, Javier Jiménez-Lamana et al. developed and optimized the SP-ICP-MS parameters, choosing the microsecond time regime and using the collision/reaction cell that allowed the background signal to be reduced to use the most selective isotope of selenium; they demonstrated the presence of SeNPs with dimensions ranging from 40 to 200 nm. This was also confirmed by TEM, where NPs around 100 nm were observed, confirming the biosynthesis process of SeNPs occurring in selenium-enriched yeast [39].

Additionally, if SeNPs are to be used in dietary supplements, there is the need to determine the composition of stabilizing capping biomolecules and also to develop fast and reliable analytical/(bio)chemical methods for the quantification of soluble Se release from the SeNPs under biologically relevant conditions. Neither of the two issues are thoroughly investigated, but they are both gaining attention within the scientific community. For example, Xu et al. investigated the composition of the capping crown of SeNPs biosynthesized by bacterium *Comamonas testosteroni* S44 using specific assays for the quantification of lipids, carbohydrates, and proteins, using SDS-PAGE combined with LC–MS/MS and proteomics for protein identification [114]. Zhang et al. investigated the stability and controlled Se release in simulated intestinal (pH 7.4), gastric (pH 1.2), and sweat media (pH 6.3), as well as free radicals in in vitro media, from NPs chemically synthesized with ascorbic acid and stabilized with chitosan [104]. They used HG-AFS for Se quantification and kinetic equations for fitting, and they found that the pH and the enzymes did not affect the stability of NPs, but the free radicals induced a high release of Se, probably by degrading the chitosan. These are interesting and relevant findings in the perspective of using SeNPs as antioxidant supplements.

Table 2 presents an overview of some of the reported techniques for Se analysis in different samples, including dietary supplements.

## 4. Conclusions

The U-shape relationship between Se status and diseases limits selenium supplementation to subjects with lower-than-optimal selenium status. The next-generation Se supplements, such as zerovalent Se nanoparticles (SeNPs) and selenized polysaccharides, are supplements with lower risk of Se excess supplementation, due to their lower toxicity, higher bioavailability, and controlled release. Zerovalent Se and Se polysaccharides slowly release active Se species through an equilibrium reaction.

One of the reasons for Se paradoxical and/or U-shaped responses is the epigenetic effect. Next-generation selenium supplements should have more balanced epigenetic effects, due to a release which is controlled by the level of the existing tissular selenium bioactive species. Moreover, next-generation Se supplements might have additional positive biological activities compared to the already known effects of Se. For example, additional effects, such as the inhibition of biofilm formation by the multi-antibiotic resistant pathogenic bacteria or stimulation of beneficial microbiome were already reported for these new forms of Se. The advantages of the advanced next-generation selenium dietary supplements will promote their consumption. The methods of analysis of next-generation selenium supplements should include a step related to chemical species separation. Such a step allows a proper characterization of the selenium forms/species, including molecular mass/dimension, and substantiates the marketing claims related to the advantages of these new selenium ingredients.

## Figures and Tables

**Table 1:** Next-generation Se species and their biological effects *.

Next-Generation Ingredients	Methods of (Bio)-Synthesizing SeNPs	Experimental Model	Biological Effect	Reference
SeNPs, chemical synthesis	(Vitamin C + Oseltamivir) (Quercetin + CdSe + ZnS), (Glutathione + NaOH)	(H1N1 influenza virus; MDCK cells), (*Escherichia coli,* *Bacillus subtilis)*, (*Staphylococcus aureus)*	Antimicrobial activity	[163,164,165]
	(L-cysteine or Ascorbic acid), (SDS + sodium sulfate × 5H_2_O or L-cysteine)	*(Pseudomonas aeruginosa aeruginosa,S.aureus)*, (*P. aeruginosa*, *Candida* spp.)	Moderate antimicrobial activity	[166,167]
	(Quercetin + CdSe + ZnS), (Se-substituted hydroxyapatite NPs)	(BGC-823 cells), (human HCCLM9 cells injected in Balb/c nude mice)	Anticancer effects	[164,168]
	Berberine-loaded Se-coated nanostructured lipid carriers	Diabetic Sprague/Dawley rats	Enhanced hypoglycemic effect	[169]
	(sodium alginate + reduced glutathione)	Male Sprague/Dawley (SD) rats	Protection against diabetic nephropathy	[170]
	Ascorbic acid + dextrin	Wistar rats	Anti-inflammatory effect in arthritis	[171]
SeNPs, biogenic synthesis	*(Enterococcus faecalis)*, (*Streptomyces minutiscleroticus)*, (*Ralstonia eutropha), (Bacillus mycoides), (Bacillus mycoides, Stenotrophomonas maltophilia)*	*(Acinetobacter* strains; type-1 dengue virus), (*S. aureus*; *B. subtilis*; *E. coli*; *P. aeruginosa)*, (*E. coli*, *P. aeruginosa*, *S. aureus, Staphylococcus pyogenes*, *Aspergillus clavatus)*, (*P. aeruginosa, S. aureus)*, (*P.* *aeruginosa*, *C. albicans)*	Antimicrobial activity	[166,167,172,173,174]
	*(Enterococcus faecalis)*	(DPPH assay; Phosphomolybdenum method)	Antioxidant effects	[172]
	*(Lactobacillus brevis)*	BALB/c mice	Anticancer effects	[175]
	*(Enterococcus faecalis)*	(Swiss albino rats)	Wound healing	[172]
SeNPs, assisted biosynthesis	BSA + ascorbic acid-assisted biosynthesis	*S. aureus*, *Staphylococcus epidermidis*, *B. subtilis*, *Klebsiella pneumoniae*	Antimicrobial activity	[176]
	BSA + glutathione-assisted biosynthesis	Male Kunming mice	Antioxidant effects	[177]
	(siRNA + vitamin C), (Polysaccharides extracted from *Dictyophora indusiata +* ascorbic acid)	(HepG-2 cell line), (HepG-2, A549, Hela, MCF-7, and PC3 cell lines)	Anticancer effects	[108,178]
	Polysaccharides from *Catathelasma ventricosum +* Ascorbic acid	Male ICR diabetic mice	Anti-diabetic activity	[109]
SeNPs, Commercial source	Not available	cashmere goats	Improved fetal growth and hair follicle development	[179]
	Not available	Boer goats	Enhanced semen and testicular GSH-Px activity, protection of the plasma membrane and mitochondria midpiece of spermatozoa	[180]
Selenized polysaccharides	Fruits of *Rosa laevigata*	SH-SY5Y neuroblastoma cells	Neuroprotective effects	[152]
	(Fruits of *Rosa laevigata)*, (*Agrocybe cylindracea)*, (*Sargassum fusiforme)*	(ABTS, DPPH, FRAP assays), (DPPH, hydroxyl radical scavenging, reducing power assays), (Kumming mice with tumor)	Antioxidant effects	[42,152,181]
	*(Agrocybe cylindracea)*	Kunming mice	Anti-ageing effects	[42]
	*(Catathelasma ventricosum)*, (Sweet potato tuber)	(Male ICR diabetic mice), (Male SD diabetic rats)	Antidiabetic effects	[182,183]
	*Hericium erinaceus*	Immature dendritic cells from ICR mice	Immunostimulant (dendritic cells maturation)	[184]
	*(Artemisia sphaerocephala)*, (Sweet potato tuber)	(HepG-2, A549, and Hela cell lines), (H22 hepatoma cell line, Female Kunming Mice)	Anti-tumor activity	[183,185]

* A549: adenocarcinomic human alveolar basal epithelial cells; ABTS: 2,2′-azino-bis(3-ethylbenzothiazoline-6-sulphonic acid); BALB/c: albino mice used in research; BGC823: gastric cancer cell line; BSA: Bovine Serum Albumin; DPPH: 2,2-diphenyl-1-picrylhydrazyl; FRAP: Ferric ion reducing antioxidant power; H22: Murine hepatoma cell line; HCCLM9: hepatocellular carcinoma cell line; HeLa: cell line derived from cervical cancer cells; Hep G-2: human hepatocyte carcinoma cell; ICR: Institute of Cancer Research; MCF-7: human breast cancer cell line from Michigan Cancer Foundation; MDCK: Madin-Darby Canine Kidney cells; PC3: prostate cancer cell line; SD: Sprague-Dawley (rat strain); SeNPs: selenium nanoparticles; SDS: sodium dodecyl sulfate; SH-SY5Y: bone marrow neuroblast cell line; siRNA: small interfering ribonucleic acid.

**Table 2:** Overview of selenium analysis and quantification methods.

Method	Samples	Advantages	Disadvantages
Inductively coupled plasma mass spectrometry (ICP-MS; in some case a collision cell was used)	Human plasma [22]; extracts of fish muscle, diets, and reference materials [245]; selenium nanoparticles [213]; radish sprouts [246]; rotifer tissue [247]; seafood [248]; glutathione peroxidase (Gpx) from bovine erythrocytes [221]; Se-rich yeast [73]; leaves, grapes, and wines [249]; mushrooms [250]	Can handle both simple and complex matrices; better detection limit than AAS and ICP-OES; small sample volume	Interference from plasma gas (Ar) and chlorides; high set-up and operational cost
Atomic fluorescence spectrophotometer (AFS)/hydride generation atomic fluorescence spectrometry (HG-AFS)	Soil samples [251]; rice [214]; Se (VI) [204]	Relatively simple equipment, the ability to analyze many samples in a short time	
Graphite furnace atomic absorption spectroscopy (GF-AAS)/flame atomic absorption spectrometry	Selenium nanoparticles [252]; pork meat [253]; sprouts of broccoli and white mustard [59]; brazil nuts [254]; peanuts [255]	High sensitivity, reduced analysis time	Matrix interference
Hydride generation atomic absorption spectrometry (HG-AAS)	Selenium nanoparticles [213], basil plants [256], meat and liver, fertilizers, and feed [257]; duck feed [258]	Minimum matrix interference	Interference of transition metals
Electrothermal atomic absorption spectrometry (ETAAS)	Cereals, milk, cheese, vegetables, fish, plasma, whole blood, and tissues [257]; dietary supplements [259]	Sensitive, high accuracy	Matrix interference in organic samples
Conjugated techniques using high-performance liquid chromatography with hydride generation atomic absorption spectrometry (HPLC–HG-AAS)	Dietary supplements [86,215]; garlic, radish sprouts, and sunflower sprouts [195]	Relatively simple	
Hydrophilic ion interaction chromatography (HILIC) with inductively coupled plasma mass spectrometric detection (ICP-MS)	Torula yeast [87]		
Ion-pairing reversed-phase liquid chromatography HPLC–ICP-MS	Fish, seafood [196]; selenomethionine and Se-methylselenocysteine in mushrooms, Se-yeast [250]		
High-performance liquid chromatography with inductively coupled plasma mass spectrometry (HPLC–ICP-MS), HPLC–Orbitrap MS	Seafood [248], leaves, grapes, and wines [249]; dietary supplements [72]	Speciation and identification of organic selenium compounds	Unknown peaks, lack of standards and reference materials
HPLC–ICP-MS and derivatization gas chromatography with atomic emission detection (GC–AED)	Dietary supplements [64]		
Ultra-performance liquid chromatography mass spectrometry (UPLC–MS/MS)	Selenomethionine in rat plasma [260]		
UV photochemical vapor generation (photo-CVG) to transform Se in its volatile species	Se (VI) [204], dietary supplements [259]		
Multidimensional chromatography with dual ICP-MS and electrospray ionization ESI-MS detection	Se-rich yeast [73]	May identify peaks that are missed by ICP-MS	Sensitive to the presence of salts
Size exclusion chromatography (SEC)	Mushrooms, Se-yeast [250]; dietary supplements [259]		
Optical emission spectrometry inductively coupled plasma (ICP-OES)	*E. durans* [93], wastewater [261]		Higher detection limit than ICP-MS; matrix interference

## Abbreviations

1D SDS-PAGE	one-dimensional sodium dodecyl sulfate polyacrylamide gel electrophoresis
A549	Adenocarcinomic human alveolar basal epithelial cells
AAS	atomic absorption spectroscopy
ABTS	2,2′-azino-bis (3-ethylbenzothiazoline-6-sulphonic acid)
AF4	asymmetrical flow field-flow fractionation
AFM	atomic force microscopy
AFS	atomic fluorescence spectroscopy
AKI	acute kidney injuring
BALB/c	albino mice used in research
BGC823	gastric cancer cell line
BMHT	betaine homocysteine methyltransferase
BSA	bovine serum albumin
CBIMMT	4-(4′-chlorobenzylideneimino)-3-methyl-5-mercapto-1,2,4-triazole
Cys	cysteine
DAD	diode array detector
DLS	dynamic light scattering
DNA	deoxyribonucleic acid
DNMTs	DNA methyltransferases
DPPH	2,2-diphenyl-1-picrylhydrazyl
DS	dietary supplements
EELS	electron energy-loss spectroscopy
EFSA	European Food Safety Authority
ESI-MS	electrospray ionization mass spectrometry
ET-AAS	electrothermal atomic absorption spectroscopy
EXAFS	Extended X-ray absorption fine structure
FAAS	flame atomic absorption spectroscopy
FFF	(a)symmetrical flow field-flow fractionation
FRAP	ferric ion reducing antioxidant power
FRAPS	fluorescence recovery after photobleaching
FTIR	Fourier-transform infrared spectroscopy
GC	gas chromatography
GF-AAS	graphite furnace atomic absorption spectroscopy
GPX	glutathione peroxidase
H22	murine hepatoma cell line
HA	humic acid
HCCLM9	hepatocellular carcinoma cell line
HDACc	histone deacetylase
HEC	hydroxyethylcellulose
HeLa	cell line derived from cervical cancer cells
Hep G2	human hepatocyte carcinoma cell
HG	hydride generation
HILIC	hydrophilic ion interaction chromatography
HPLC–HG-AAS	high-performance liquid chromatography hydride generation atomic absorption spectrometry
HR-CS	high-resolution continuous source
ICP-MS	inductively coupled plasma mass spectrometry
ICP-OES	inductively coupled plasma optical emission spectrometry
ICP-TOF-MS	inductively coupled plasma time-of-flight mass spectrometry
ICR	Institute of Cancer Research
IEF	isoelectric focusing electrophoresis
INAA	instrumental neutron activation analysis
iSE	inorganic selenium
LA-ICP-MS	laser ablation inductively coupled plasma mass spectrometry
LC–MS	liquid chromatography–mass spectrometry
LOD	limit of detection
MALS	multiple-angle light scattering
Met	methione
SeMet	selenomethionine
MeSeCys	methylselenocysteine
MeSeCys	methylselenocysteine
MCF-7	human breast cancer cell line from Michigan Cancer Foundation
MDCK	Madin-Darby Canine Kidney cells
MP-AES	microwave plasma atomic emission spectrometry
MS	mass spectrometry
MSeA	methylseleninic acid
NOAEL	no observed adverse effect level
NPs	(selenium) nanoparticles
OTC	over-the-counter
PAGE	polyacrylamide gel electrophoresis
PC3	prostate cancer cell line
photo-CVG	photochemical vapor generation
RDA	recommended daily allowance
SD	Sprague/Dawley (rat strain)
SDS	sodium dodecyl sulfate
SeCys	selenocysteine
SeCys_2_	selenocystine
SEC	size-exclusion chromatography
SeCys	selenocysteine
SeHLan	selenohomolanthionine
SEM	scanning electron microscopy
SeMet	selenomethionine
SeNPs	selenium nanoparticles
SH-SY5Y	bone marrow neuroblast cell line
siRNA	small interfering ribonucleic acid
SPs	selenized polysaccharides
SP-ICP-MS	single-particle inductively coupled plasma mass spectrometry
TEM	transmission electron microscopy
TrxR	thioredoxin reductase
TXRF	total reflection X-ray fluorescence spectroscopy
UL	tolerable upper limit
US	Unites States
U.S. EPA	United States Environmental Protection Agency
UV-PVG	ultraviolet photochemical vapor generation system
XANES	X-ray absorption near-edge structure
XFA	X-ray fluorescence analysis
XRD	X-ray diffraction
